# Utilization of machine learning for dengue case screening

**DOI:** 10.1186/s12889-024-19083-8

**Published:** 2024-06-11

**Authors:** Bianca Conrad Bohm, Fernando Elias de Melo Borges, Suellen Caroline Matos Silva, Alessandra Talaska Soares, Danton Diego Ferreira, Vinícius Silva Belo, Julia Somavilla Lignon, Fábio Raphael Pascoti Bruhn

**Affiliations:** 1 Laboratory of Veterinary Epidemiology, Postgraduate Program in Veterinary, Federal University of Pelotas (UFPel), Capão do Leão, RS Brazil; 2 Automation Department, Federal University of Lavras, Lavras, Minas Gerais Brazil; 3 Laboratory of Veterinary Epidemiology, Graduate Program in Microbiology and Parasitology, Federal University of Pelotas, Capão do Leão, Rio Grande do Sul Brazil; 4 Federal University of São, João del-Rei, Midwest Dona Lindu campus, Divinópolis, Minas Gerais Brazil; 5 Laboratory of Veterinary Epidemiology, Preventive Veterinary Department, Federal University of Pelotas,, Capão do Leão, Rio Grande do Sul Brazil

**Keywords:** Arboviruses, Artificial intelligence, Clinical signs, Healthcare systems

## Abstract

Dengue causes approximately 10.000 deaths and 100 million symptomatic infections annually worldwide, making it a significant public health concern. To address this, artificial intelligence tools like machine learning can play a crucial role in developing more effective strategies for control, diagnosis, and treatment. This study identifies relevant variables for the screening of dengue cases through machine learning models and evaluates the accuracy of the models. Data from reported dengue cases in the states of Rio de Janeiro and Minas Gerais for the years 2016 and 2019 were obtained through the National Notifiable Diseases Surveillance System (SINAN). The mutual information technique was used to assess which variables were most related to laboratory-confirmed dengue cases. Next, a random selection of 10,000 confirmed cases and 10,000 discarded cases was performed, and the dataset was divided into training (70%) and testing (30%). Machine learning models were then tested to classify the cases. It was found that the logistic regression model with 10 variables (gender, age, fever, myalgia, headache, vomiting, nausea, back pain, rash, retro-orbital pain) and the Decision Tree and Multilayer Perceptron (MLP) models achieved the best results in decision metrics, with an accuracy of 98%. Therefore, a tree-based model would be suitable for building an application and implementing it on smartphones. This resource would be available to healthcare professionals such as doctors and nurses.

**Supplementary Information:**

The online version contains supplementary material available at 10.1186/s12889-024-19083-8.

## Introduction

Dengue is the most important arbovirus transmitted by mosquitoes (mainly *Aedes aegypti*) in humans and is considered a reemerging disease with significant impacts on global public health, particularly in Asian and Latin American countries [1, 2]. It is caused by infection with any of the four known serotypes of the *Flavivirus* genus (DENV-1 to DENV-4) and can result in a wide spectrum of clinical manifestations, ranging from asymptomatic to severe cases. Symptoms include fever, nausea, vomiting, skin rashes, and muscle pain, which can progress to bleeding and death [3–5]. However, some of these clinical signs are similar to those of other illnesses and may hinder the adoption of appropriate clinical management, consequently predisposing individuals to severe forms [5].

The exact incidence of dengue is difficult to determine; however, it is estimated that the number of annual infections varies between 284 and 528 million worldwide [6], with approximately 100 million symptomatic infections and 10,000 deaths annually [7, 8]. According to the Pan American Health Organization (PAHO) [9], the number of cases rose to 16.2 million in the last decade (2010–2019) in the Americas. The year with the highest number of cases recorded on the American continent was 2023, with a total of 4,565,911 cases, including 7,653 serious cases and 2,340 deaths. This situation of high transmission persists in 2024. In Brazil, the number of cases reported in the first 12 epidemiological weeks of 2024 was 2,966,339, representing an increase of 227% compared to the same period in 2023 and an increase of 284% compared to the average of the last five years in the country [9]. Furthermore, according to PAHO, Brazil is the most affected country on the American continent, representing 83% of cases [9], further increasing the risk of future epidemics [10, 11].

The disease also imposes substantial economic, social, and political burdens worldwide, with millions of people affected each year, and its incidence has been increasing over the past 50 years [12]. Despite receiving greater public health investments compared to other infectious diseases, dengue remains on the World Health Organization (WHO) list of neglected tropical diseases [13].

Dengue cases can be confirmed through clinical-epidemiological or laboratory evaluation [5], including virus isolation, molecular tests, and serological assays, depending on the stage of the disease [3]. Early recognition of the disease contributes to reducing morbidity and mortality by allowing suspected patients to receive faster access to supportive treatment and appropriate medical monitoring [14, 15]. Therefore, the development of an intelligent system to detect dengue cases early is crucial for the favorable evolution of the disease, especially to countries with high incidence, such as Brazil [14]. In Brazil, epidemiological surveillance systems lack easily applicable tools for efficient patient screening and optimization of medical care. An efficient patient screening can provide early dengue detection cases, which leads to the optimization of health expenses with considerable savings. A machine learning-based model based on a combination of characteristic disease symptoms may be useful for characterizing dengue fever and guiding clinical investigation [16, 17]. Machine learning has been used in various research areas, yielding satisfactory results for healthcare services [14, 18, 19]. The models built from machine learning techniques are capable of “learning” from data, and identifying the most relevant attributes for the application. Machine learning models utilize optimizer algorithms for the training task. After this process, the model becomes capable of classifying patterns, grouping data into similar sets, or predicting values with acceptable accuracy [20].

Machine learning has been employed for clinical diagnosis in various diseases, including vector-borne infections [19, 21–24]. However, most research has focused on predicting the evolution of dengue using laboratory data, while other studies have used the technique to evaluate the dynamics of disease transmission [23]. Thus, studies involving the use of machine learning to screen dengue cases using clinical data are considered incipient, highlighting the need for research that effectively contributes to the assessment of clinical signs and symptoms to assist medical decision-making, thereby reducing the waiting time for clinical care in urban centers [24, 25].

This study aimed to identify important variables for conducting the screening of dengue cases using clinical data through machine learning techniques and evaluate the accuracy of the constructed models. As a final result, we hope the developed model may be easily implemented on a mobile app to be used by healthcare professionals.

## Data and methods

This is quantitative research conducted with secondary data obtained from individual dengue notification forms through the National Notification System for Diseases (SINAN) [26]. Data from the Brazilian states of Minas Gerais/MG and Rio de Janeiro/RJ were analyzed, both of which had a high number of reported cases in the years 2016 and 2019. This database is available in DataSUS [27] and is fully anonymized and contains information on sex, age, race, place of residence, clinical signs, diagnosis, confirmation criteria, and case outcomes.

The study was approved by the Ethics Committee of the Faculty of Medicine of the Federal University of Pelotas, CAAE 46019321.6.0000.5317, in accordance with all ethical principles and current legislation for research involving human beings.

The model design was conducted according to the following stages: (i) preprocessing (data integration and organization; and variable normalization); (ii) feature selection; (iii) model training; and (iv) model evaluation.

### Data set and preprocessing

The data for this study were retrieved from SINAN (https://portalsinan.saude.gov.br/) and consolidated to only include complete case records. The variables related to laboratory diagnosis were also excluded from the data set since the aim of the study was to use machine learning to assist in medical decision-making based only on clinical data. Finally, the data set consisted of 23 attributes (variables) that, except for age, were categorized as 0 (absence) and 1 (presence).

According to the dengue manual [5], a suspected case of dengue is defined as any patient with acute fever accompanied by two or more symptoms such as headache, retro-orbital pain, myalgia, arthralgia, prostration, or rash, with or without the presence of bleeding. During epidemic years, the diagnosis can be made clinically and epidemiologically based on the patient’s medical history and the presence of clinical signs. Laboratory diagnosis is performed through virus isolation, molecular tests, and serological assays, depending on the stage of the disease.

The final data set consisted of 229,113 positive cases of dengue and 135,163 negative cases of dengue. All data were confirmed by laboratory tests. To balance the data set, clean and organized it, and due to computational limitations, a random selection of 10,000 positive cases and 10,000 negative cases of dengue was performed. This ensured that there was no majority class biasing the model classification.

#### Data normalization

All variables of the data set were standardized according to the standard score (z-scores) normalization, following Eq. (1).

1 $${x}_{i,norm}=\frac{{x}_{i}-{\mu }_{i}}{{\sigma }_{i}}$$

where, *x*_*i*,*norm*_ is the normalized version of variable *i* (*x*_*i*_), *µ*_*i*_ and *σ*i are the mean and standard deviation values of variable *i*, respectively.

### Feature selection

For feature selection and machine learning model training, the Python programming language, version 3.8, was used in conjunction with the Scikit-Learn, Pandas, Numpy, and Matplotlib libraries [28–32]. These are open-source libraries with various features for data analysis and mining, statistical modeling, and supervised and unsupervised learning.

Variable selection was performed using the mutual information (MI) technique [28, 33]. MI is a natural measure of the dependence between random variables. It is always nonnegative, and zero if and only if the variables are statistically independent. Mutual information takes into account the whole dependence structure of the variables, and not just the covariance, like principal component analysis (PCA) and related methods [33]. This definition is useful within the context of feature selection because it gives a way to quantify the relevance of a feature subset with respect to the output vector [34]. This method is effective in removing variables with low relevance, simplifying the data, and improving model performance [35, 36]. In this study, the MI was applied to measure the statistical dependence between the input variables and the output variable. Mutual information may be calculated by Eq. (2):

2 $$\eqalign{& {\rm{I}}\left( {x;y} \right) = \mathop \sum \limits_{i = 1}^n \mathop \sum \limits_{j = 1}^n p\left( {x\left( i \right),y\left( j \right)} \right) \cdot \cr & log\left( {{{p\left( {x\left( i \right),y\left( j \right)} \right)} \over {p\left( {x\left( i \right)} \right) \cdot p\left( {y\left( j \right)} \right)}}} \right) \cr}$$

where MI is zero when *x* and *y* are statistically independent, i.e., *p*(*x*(*i*)),*y*(*j*)) = *p*(*x*(*i*))*p*(*y*(*j*)). *p*(*x*,y) is the joint probability of *x* and *y*, and p(x) and p(y) are the marginal probabilities.

### Model training

In the stage of constructing predictive models, the target attribute (or class), namely the diagnostic attribute for Dengue, was assigned. The classification models tested were decision tree, K-nearest neighbors (KNN), logistic regression, and Multilayer Perceptron Neural Network (MLP). These machine learning models are available in the Scikit-learn library.

**Fig. 1:**
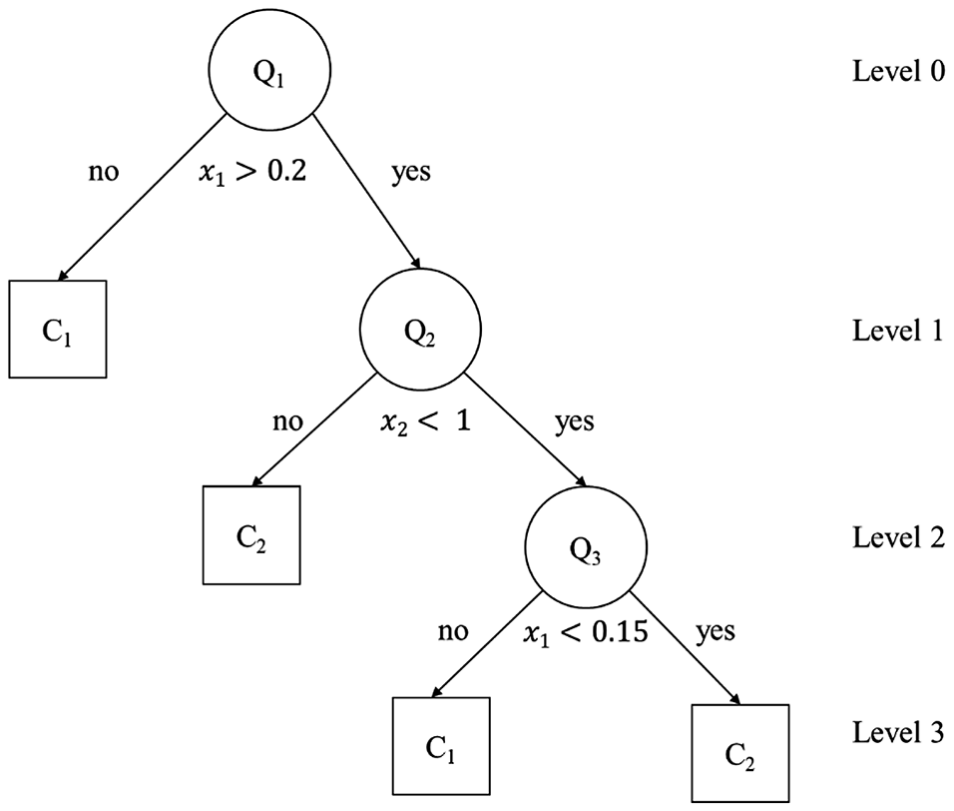
Example of a decision tree with three levels. Q1, Q2 and Q3 represent the nodes, and the squares containing the output class (C1 or C2) are the leaves

Decision trees are models suitable for solving classification problems into classes or categories. The operational flowchart of a decision tree is based on the construction of rules, with responses generated based on the input attributes (questions). In these tree structures, leaves represent class labels and branches represent conjunctions of features that lead to those class labels [37]. Decision tree learning utilizes a divide-and-conquer strategy, employing a greedy search to identify optimal split points within a tree structure. This splitting process iterates recursively from the top-down until the majority, or all, of the records are classified under specific class labels. Figure 1 shows an example of a decision tree with three levels.

**Fig. 2:**
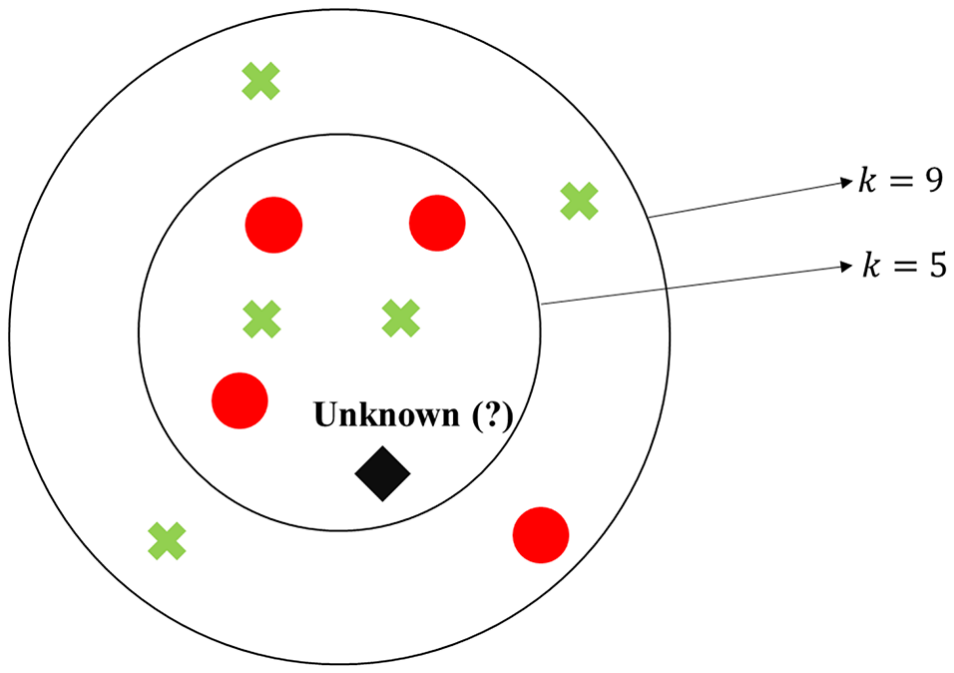
Example of the KNN classifier strategy in the classification of an unknown data considering *k* = 5 and *k* = 9

K-nearest neighbors (KNN) is a classifier that looks for data close (similar) to each other. KNN takes advantage of not performing any prior training like other classifiers [38]. In the operational phase, the distances between the test data and the stored data points are measured. Then, the *k* nearest data points are counted, and the class that has the highest number of nearest neighbors within the selected *k* is assigned to the test data [39]. Figure 2 illustrates this strategy, where the unknown data is classified as red class for *k* = 5, but if *k* = 9 is considered, the unknown data is assigned as green class. This example is interesting to show that the definition of parameter *k* is crucial.

**Fig. 3:**
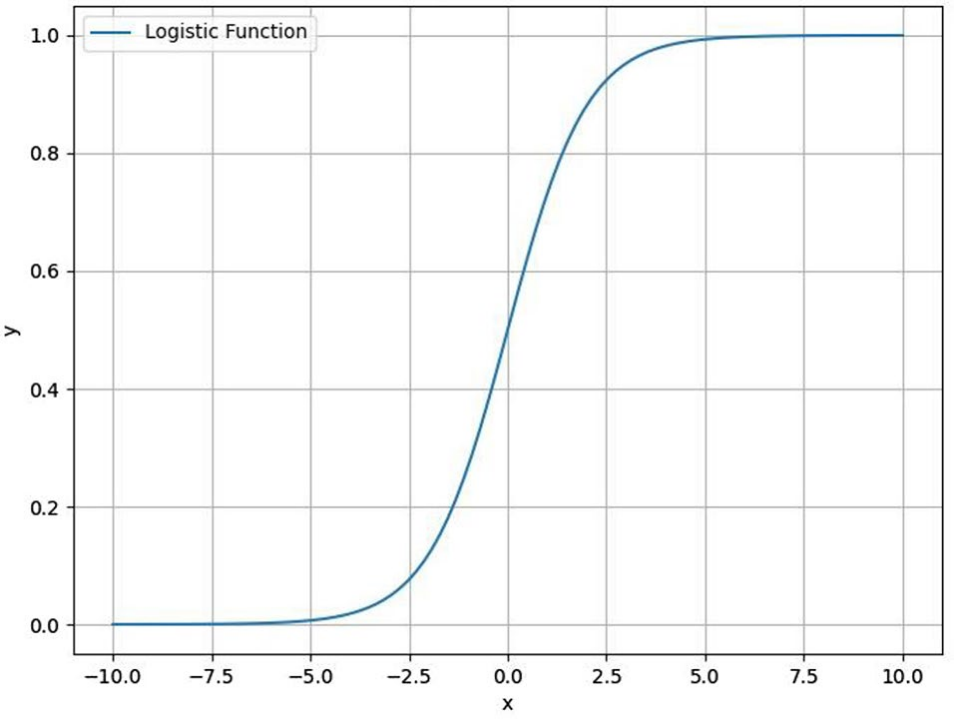
Sigmoid function. Variables *x* and *y* refer to the input and output values of the function, *y* = *f*(*x*)

The Logistic regression model performs an approximation of the posteriori probability and its discriminant function is calculated by the sigmoid function applied to a linear model [40]. Rather than modeling response variable directly, Logistic regression models the probability of the response variable belongs to a particular category (class) [40]. Sigmoid Function is a mathematical function used to map the predicted values to probabilities (see Fig. 3). The function has the ability to map any real value into another value within a range of 0 and 1. The rule is that the value of the logistic regression must be between 0 and 1. Thus, a threshold value is used to define the probability of either 0 or 1. Considering a case of two classes (A and B), values above the threshold value tend to 1, classifying the unknown data as class A, and values below the threshold value tend to 0 (class B).

**Fig. 4:**
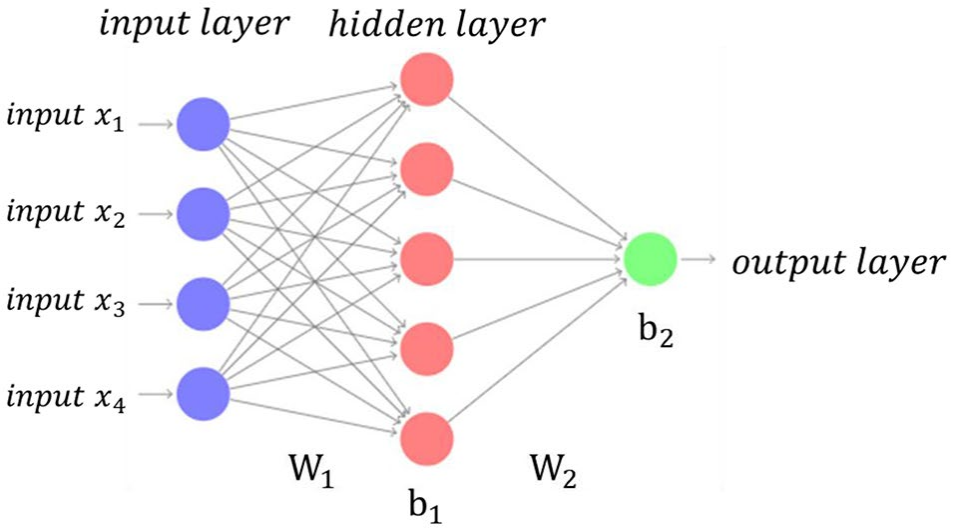
Example of a multi-layer perceptron with one hidden layer. W_1_ and W_2_ represent the weight matrix of the first and hidden layers. b_1_ and b_2_ represent the bias vectors of the first and hidden layers

Finally, the Multilayer Perceptron (MLP) is a neural network model in which the neurons of the model are divided into layers. They are models with good generalization capacity and the ability to perform nonlinear mapping between input and output data. Their training is done through the error backpropagation algorithm [41, 42]. Figure 4 shows an example of a multi-layer perceptron with two layers, four inputs (*x*_1_, *x*_2_, *x*_3_ and *x*_4_), five neurons in the hidden layer, and one neuron in the output layer. After training, the operational phase of this MLP is described by Eq. (3):

3 $$y=f\left(g\left({\mathbf{x}}^{\text{T}}{\mathbf{W}}_{1}+{\mathbf{b}}_{1}\right){\mathbf{W}}_{2}+{\mathbf{b}}_{2}\right)$$

where *y* is the output of the MLP, x = [*x*_1_* × *_2_* × *_3_* × *_4_]^*T*^ is the input vector, **W**_1_ and **W**_2_ are the weight matrices with dimensions 4 × 5 and 5 × 1, respectively, and **b**_1_ and **b**_2_ are the bias vectors with dimensions 1 × 5 and 1 × 1, respectively. Considering a case of two classes (A and B), for a sigmoid function in the output layer (which was the case of this work), if *y* > 0.5 the input data is classified as class A, otherwise it is classified as class B. The weight matrices **W**_1_ and **W**_2_, and the bias vectors **b**_1_ and **b**_2_ are adjusted during training by the backpropagation algorithm.

### Experimental setup and model evaluation

Before performing the prediction of the models, the dataset was divided into two parts: training (70%) and testing (30%). To perform the training, the k-fold cross-validation technique was used, with k = 10. This technique contributes to generating a more robust model with less bias and/or overfitting tendencies. It is a method that uses a portion of the data for model training and performs validation (testing) by challenging the classifiers to find the solution with the inclusion of new data [43]. In this way, the dataset was divided into partitions (folds), and the model was trained on all but one (k-1) of the data sets. Next, the model was evaluated on the dataset that was not used for training. This process was repeated 10 times, with a different subset reserved for evaluation each time (and excluded from training). Thus, the dataset used for final testing is not used during cross-validation, providing new data to the classifiers.

During the cross-validation process, the hyperparameters of the classifiers were varied according to Table 1. The hyperparameters that presented the best predictive results are displayed in Table 2.

**Table 1:** Hyperparameter range used during the classifiers training

Model	Hyperparameter	Value range
Decision Tree	Max depth	From 2 to 10
	Split criterion	Entropy Gini Index
MLP	Hidden layer size	From 2 to 15
	Activation Function	Hyperbolic tangent Logistic ReLu
Knn	Number of neighbors	3, 5, 7, 9
Logistic Regression	Regularization	L2 L1 Elasticnet

**Table 2:** Hyperparameters with the best results

Model	Hyperparameter	Value
Decision Tree	Max depth	5
	Split criterion	Entropy
MLP	Hidden layer size	5
	Activation Function	Hyperbolic Tangent
Knn	Number of neighbors	3
Logistic Regression	Regularization	L2

The metrics applied in the training data set were presented in mean ± standard deviation, corresponding to the 10 model executions of the k-fold. For the test data set, the results corresponding to the application of the model in the new data.

After constructing the models, the performance of the classifiers was evaluated. The confusion matrix (Table 3) was used to assess the performance of the classification models. For binary problems, the size of the confusion matrix is 2 × 2, as shown in Table 3. From the confusion matrix, it is possible to calculate several metrics to evaluate classifier models, as it can be seen in Table 4.

**Table 3:** Confusion matrix used for calculating the evaluation metrics of the machine learning models

		Predicted Class	
		Positive (Dengue)	Negative (Non-Dengue)
True Class	Positive (Dengue)	TP	FP
	Negative (Non-Dengue)	FN	TN

Where

TP = true positive

FP = false positive

FN = false negative

TN = true negative

**Table 4:** Metrics for classifier evaluation used in this work

Measure	Formula
Accuracy, recognition rate	$\frac{TP+TN}{TP+TN+FP+FN}$
Sensitivity, true positive rate, TP, recall	$\frac{TP}{TP+FN}$
Specificity, true negative rate	$\frac{TN}{TN+FP}$
Precision	$\frac{TP}{TP+FP}$
F, F1, F-score, harmonic mean of precision and recall	${\eqalign{& 2\, \times \,precision \cr & \times \,recall \cr} \over \eqalign{& precision \cr & \, + \,recall \cr} }$

The performance of the models was also evaluated using the receiver operating characteristic (ROC) curve, which represents the relationship between sensitivity and specificity. The performance of the model is evaluated by the area under the curve (AUC), where a higher AUC (closer to 1) indicates better performance. After training and testing, a graph with the ROC curve was constructed, plotting the results of all models. This allowed for visualizing the performance of the models used.

## Results

**Fig. 5:**
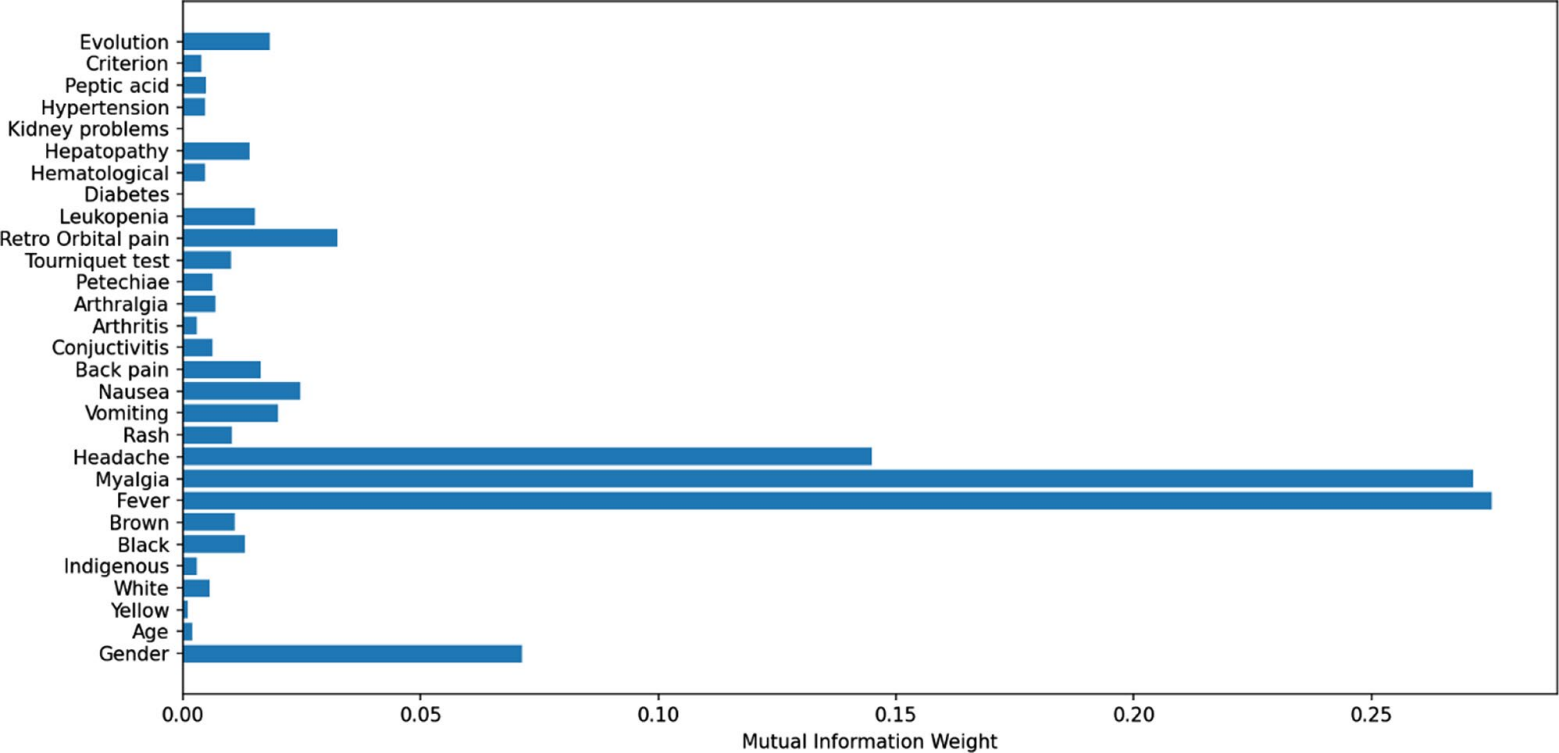
Relationship of independent variables in the database with the outcome variable (confirmed or discarded case)

In the years 2016 and 2019, the states of Minas Gerais and Rio de Janeiro collectively reported over 882,612 notifications of suspected dengue cases. Out of these, 229,113 (21.21%) corresponded to confirmed cases, and 135,163 were cases discarded based on laboratory criteria. During the same period, 645,264 cases were confirmed, and 186,248 cases were discarded based on clinical epidemiological criteria. In 8,235 notifications, the confirmation field was either blank or filled incorrectly. The mutual information criterion was used to assess the relationship between the 23 independent variables related to clinical diagnosis and the dependent variable (confirmed or discarded dengue cases) (Fig. 5). A series of 10 tests were conducted, and the model with 10 variables exhibited the most favorable performance based on evaluation metrics. Consequently, this model was chosen for training and testing. The selected variables included gender, age, fever, myalgia, headache, vomiting, nausea, back pain, exanthema, and retro-orbital pain.

**Fig. 6:**
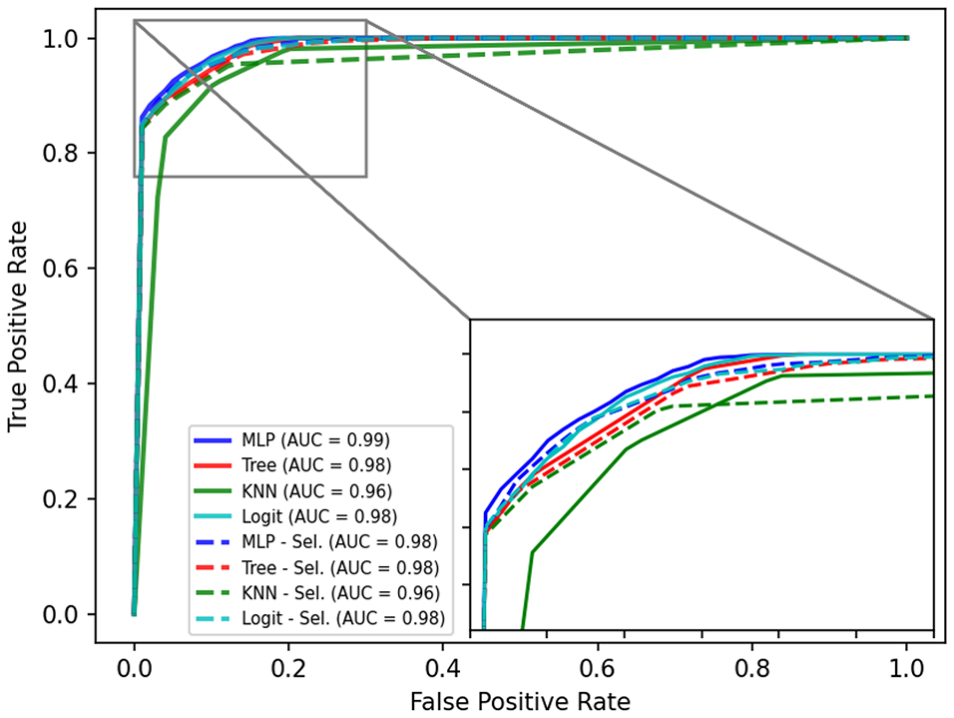
Representation of classifier performance through the ROC curve. Mlp – Multilayer Percepton; Knn - K-nearest neighbors

Figure 6 illustrates the model performance through the ROC curve. The ROC curve was built for all applied classifiers considering two situations: using all of the input variables and using only the variables selected by MI. Taking into account the practical application of the method (usability), it is suggested the use of the models that considered only the selected variables, since they will be used by healthcare professionals daily. Thus, the following performance tables refer to the models designed using only the selected variables.

The tested techniques yielded satisfactory results, with accuracy values above 90%. The metric values from the k-fold during model training are shown in Table 5. The metrics values for the k-fold execution are displayed in format mean ± standard deviation. The decision tree and MLP were the models with the best performance. Table 6 displays the evaluation metric values for the test data set. It can be observed that the decision tree achieved the best values of ACC and F1, while the MLP demonstrated the best performance in the AUC metric.

**Table 5:** Results of the evaluation metrics for the k-fold model in dengue case screening

Model	Accuracy (ACC)	F1-Score	AUC*
Decision Tree	0.9254 ± 0.0057	0.9283 ± 0.0053	0.9843 ± 0.0013
Knn**	0.9206 ± 0.0040	0.9231 ± 0.0040	0.9672 ± 0.0044
MLP***	0.9289 ± 0.0050	0.9298 ± 0.0049	0.9862 ± 0.0016
Logistic Regression	0.9256 ± 0.0049	0.9270 ± 0.0048	0.9853 ± 0.0018

* AUC: Area under the curve; ** Knn: K-nearest neighbors; ***MLP: Multilayer Perceptron

**Table 6:** Results of evaluation metrics for machine learning test models used for dengue case screening

Model	Accuracy (ACC)	F1-Score	AUC*
Decision Tree	0.9252	0.9283	0.9853
Knn**	0.9223	0.9251	0.9641
MLP***	0.9313	0.9333	0.9878
Logistic Regression	0.9312	0.9323	0.9874

* AUC: Area under the curve; ** Knn: K-nearest neighbors; ***MLP: Multilayer Perceptron

## Discussion

This study aimed to assess the clinical variables that can aid in training machine learning models for dengue cases screening. This methodology was devised to enhance the classification of potential cases, thereby reducing waiting times for medical attention in densely populated urban centers and addressing underreporting in remote areas with limited or absent healthcare resources.

The obtained results are highly relevant in terms of public health. The classification model’s predictive efficacy utilizing patients’ clinical data, accessible via medical histories and rapid clinical tests, demonstrated satisfactory performance, indicative of its potential integration as a valuable tool within healthcare services. Another positive aspect of these findings is the utilization of binary data in the model (yes or no) and a small number of variables, which simplifies its application for healthcare professionals.

Based on the ROC curves displayed in Fig. 6, all classifiers performed slightly better without feature selection, except the Knn Classifier. The MLP model achieved the best AUC (AUC = 0.99), followed by the Tree and Logistic classifiers (AUC = 0.98) and the Knn (AUC = 0.96). Considering the ACC and F1-Score metrics, the MLP and Logistic classifiers achieved the best results for testing data (see Table 6). Regarding the AUC metric, the MLP neural network, Logistic Regression and Decision Tree achieved similar results, with differences in the third and fourth decimal place (see Table 6). Considering the low computational complexity during the operational phase of the decision tree and its explainable capacity, the decision tree model is the recommended one for the screening of dengue cases.

A study conducted by Tanner et al. [16] utilized a decision tree model to screen dengue cases in Singapore and Vietnam using complete blood count data, achieving an AUC value of 88%. The authors reported that they selected the model because decision algorithms are easy to apply and understand, and they handle missing data effectively. However, despite these promising results, the use of laboratory data poses a limiting factor for model implementation, particularly in countries like Brazil, which experience a high incidence of dengue and a scarcity of healthcare resources. Conducting laboratory tests for all suspected cases is unfeasible. Consequently, by utilizing real data from notification forms in the SINAN system, which are generated at the time of medical consultation and finalized only after the outcome is determined, it was possible to train a classification model with satisfactory predictive performance. Vasconcelos Silveira et al. [24] used the 42 variables available in the notification form to train machine learning models for the prediction of three arboviruses transmitted by Aedes aegypti and found that the Random Forest, which is similar to a decision tree model, model achieved the best classification results (90.64%). Decision algorithms are easy to apply and understand, in addition to handling missing data effectively, and they have shown promising results in the cited studies and in the present study [44].

Other tools have been used for case screening, and an example of the expansion of these strategies was seen during the Covid-19 pandemic. The need for tools that streamline case screening and risk analysis became evident, leading to an increased use of online tools and the development of mobile applications. Therefore, the use of high-quality and complete data for training and validating the models before their deployment for medical use has become essential [25, 45, 46], since low-quality data can generate poor classification results and an inconsistent model.

Thus, the model evaluation considered the best results in the evaluation metrics. The decision to assess these metrics stems from the fact that the study aimed to build a model that assists in the screening of suspected cases; therefore, it is important for the model to have high sensitivity values to minimize the chance of dismissing a potential dengue case.

Although the study yielded satisfactory outcomes from the trained models, it is important to acknowledge certain limitations inherent to this research. One significant limitation arises from the fact that the models were trained exclusively using data from two specific municipalities, rather than encompassing a broader national dataset. Given the considerable regional diversity within Brazil, it is plausible that the model’s accuracy could be affected when extended to encompass data from other locations. Consequently, the need for further studies becomes apparent, with the aim of refining and expanding upon these findings.

Furthermore, another notable limitation is linked to the reliance on secondary data sources. This reliance introduces a potential loss of information due to various factors, including incomplete forms, potential oversight of clinical signs within records, and the inability to incorporate additional variables. For instance, the consideration of a patient’s history of virus exposure remains unaccounted for in the current study.

However, despite these limitations, machine learning was efficient in case screening, making it a potential tool for implementation in healthcare services. It is worth mentioning that the designed tree-based model is computationally simple, since it uses only 8 inputs, 10 leaf nodes, and 8 decision nodes, performing thus, at worst case, 5 operations to process information of one patient. Therefore, the proposed tree-based model is suitable for implementation in Apps for smartphones and can be performed in computers with basic configurations.

The screening model developed in this study aims to provide valuable assistance to healthcare professionals in the identification of dengue cases through the utilization of clinical variables. The early diagnosis of a case holds the potential to facilitate timely patient monitoring, aiding in the identification of severe cases and enabling the prompt initiation of supportive treatment. This proactive approach may play a pivotal role in preventing disease progression or fatalities.

It is important to emphasize that the classification model is not designed to replace medical care. Instead, its primary purpose is to assist in the selection of suspected cases for comprehensive medical evaluation, thereby potentially alleviating the strain on healthcare services by streamlining patient management.

## Conclusions

This paper aimed to present an application of machine learning techniques for screening dengue cases. The utilization of feature selection has diminished the number of input variables for the classification model, pinpointing the most significant variables in the screening of dengue cases. This is of paramount importance, given that the application will be employed daily by healthcare professionals, where a simplified system allows for an enhanced user experience and ease of application adoption.

Among the employed classifies, the decision tree model obtained the best compromise between predictive performance and computational complexity. Thus, one recommends its implementation in mobile applications or on computers with basic configurations to prevent disease progression. The use of secondary data reinforces the importance of properly filling out official disease notification forms by these professionals. Still, the need for more studies stands out, with the aim of refining and expanding these findings, such as the scope of national datasets from other locations and the influence of the number of variables used.

### Electronic supplementary material

Below is the link to the electronic supplementary material.

Supplementary Material 1

## Data Availability

All data generated or analyzed during this study are available within the article and its supplementary information files.
